# Clinical Factors Influencing Improvement in Disorganization in Clinical High‐Risk for Psychosis: Preliminary Findings From a 2‐Year Follow‐Up

**DOI:** 10.1111/appy.70021

**Published:** 2026-04-08

**Authors:** Lorenzo Pelizza, Emanuela Leuci, Emanuela Quattrone, Michele Occhionero, Arianna Biancalani, Derna Palmisano, Simona Pupo, Giuseppina Paulillo, Clara Pellegrini, Pietro Pellegrini, Marco Menchetti

**Affiliations:** ^1^ Department of Biomedical and Neuromotor Sciences Alma Mater Studiorum Università di Bologna Bologna (BO) Italy; ^2^ Department of Mental Health Azienda USL di Parma Parma (PR) Italy; ^3^ Pain Therapy Service, Department of Medicine and Surgery Azienda Ospedaliero‐Universitaria di Parma Parma (PR) Italy

**Keywords:** clinical high risk for psychosis, disorganization, early intervention

## Abstract

Disorganization is under‐treated in early psychosis. We aimed at exploring clinical factors potentially improving disorganization in individuals at Clinical High Risk for Psychosis (CHR‐P) across 2 years of follow‐up. About 180 participants completed the GAF and the PANSS. Longitudinally, 111 (61.7%) individuals had a decrease in disorganization. Significant associations of this improvement were with shorter duration of untreated illness, higher improvement in negative symptoms, and antipsychotic prescription.

## 1 Introduction

“*Disorganization*” is considered a “core” symptom in psychosis (Bleuler 1911). However, it received *less attention* than positive and negative symptoms, due to its incorporation within the positive domain. This conflation was replicated into the assessment scales for psychosis psychopathology (e.g., the PANSS) and the current measures of disorganization have been extrapolated using factor analysis.

Increasing evidence showed that disorganization is a predictor of *poor prognosis* in Clinical High Risk for Psychosis (CHR‐P), more than positive and similarly to negative symptoms (Prasad et al. 2023). First‐line therapies in CHR‐P protocols should include disorganization as a psychopathological target. However, empirical investigations about disorganization in CHR‐P subjects are limited, especially longitudinal ones. Specifically, we previously reported a longitudinal improvement in disorganization in CHR‐P individuals (AAA 2025). The *aim* of this research was to further explore any associations of this improvement in disorganization with treatment response and clinical/sociodemographic characteristics in CHR‐P individuals treated in an Italian CHR‐P program across 2 years of follow‐up.

## 2 Methods

All participants were recruited from the “Parma At‐Risk Mental States” (*PARMS*) protocol (Italy) between January 2016 and December 2021 (AAA 2023). *Inclusion criteria* were age 12–25 years, help‐seeking request, and to meet CHR‐P criteria as defined in the “Comprehensive Assessment of At‐Risk Mental States”. *Exclusion criteria* were previous exposure to antipsychotics (AP), previous psychotic episodes, medical condition with psychiatric symptoms, and known intellectual disability.

Based on the course of disorganization, we created two different subgroups: With and without improvement in disorganization (measured with the PANSS “Disorganization” factor scores) (CHR‐P/ID+ vs. CHR‐P/ID‐). A 30% decrease in disorganization at follow‐up was used as a common benchmark for clinically meaningful improvement in PANSS scores (Lin et al. 2018). We preferred to split the total sample according to improvement versus no improvement in disorganization to enhance any potential contrast in treatment response between the subgroups, with a preliminary purpose of examining which treatments contributed most to it. In the clinical assessment, we used the “Global Assessment of Functioning” scale (American Psychiatric Association 2013) and the PANSS (see Supporting Information [Tables S1 and S2] for details about the clinical assessment used in our research). The *DSM‐5 diagnosis* was made at entry by two trained PARMS staff members with the SCID‐5 (Structured Clinical Interview for DSM‐5 disorders) (First et al. 2016). All data were collected at presentation and every 12 months during the follow‐up. CHR‐P individuals were provided with a *multi‐professional team* for early intervention (see Supporting Information [Table S3] for details).

Inter‐group comparisons were explored with the Chi‐square or the Mann–Whitney U test. In the CHR‐P/ID+ subgroup, multiple linear regression analyses, and Spearman's correlation coefficients were used to investigate longitudinal association of improvement in disorganization with clinical/sociodemographic parameters. Specifically, we calculated the deltas between T0 and T1/T2 PANSS scores as primary clinical variables to explore over time because they better described the longitudinal change of psychopathology (Ver Hoef 2012).

## 3 Results

At baseline, 180 CHR‐P were enrolled (see Supporting Information [Table S4] for details on their clinical/sociodemographic features): of them, 111 (61.7%) were included in the CHR‐P/ID+ subgroup. CHR‐P/ID+ individuals had shorter “Duration of Untreated Illness” (DUI) (38.89 ± 40.01 VS 58.42 ± 58.59; z = −2.344; *p* = 0.019; *r* = 0.107) and lower baseline PANSS “Affect” score (14.45 ± 5.19 VS 16.91 ± 4.86; z = −2.736; *p* = 0.009; *r* = 0.279). Across the follow‐up (see Supporting Information [Figure S1] for details), longitudinal improvement in disorganization in CHR‐P/ID+ individuals was positively correlated with reduction in PANSS “Negative Symptoms” (ρ = 0.520; *p* = 0.001; z = 0.590), “Affect” (ρ = 0.369; *p* = 0.001; z = 0.401), and “Resistance/Excitement‐Activity” (ρ = 0.296; *p* = 0.009; z = 0.324) scores, while was negatively correlated with DUI (ρ = −0.275; *p* = 0.032; z = .‐236) (see Supporting Information [Table S5] for details). It was also positively associated with reduction in PANSS “Uncooperativeness” (ρ = 0.232; *p* = 0.039; z = 0.161) and “Lack of Judgment/Insight” (ρ = 0.226; *p* = 0.023; z = 0.123) subscores.

In the CHR‐P/ID+ subjects, the longitudinal decrease in disorganization was predicted at baseline by AP equivalent dose (partial R^2^ = 0.117), shorter DUI (pR^2^ = 0.110), and lower severity in negative symptoms (pR^2^ = 1.88), and across the follow‐up by the total number of psychoeducational sessions provided (pR^2^ = 0.090) and the longitudinal improvement in negative symptoms severity (partial R^2^ = 0.124) (Table 1).

**TABLE 1:** Improvement in PANSS “Disorganization” factor scores and its associations with clinical parameters and specialized treatment components of the PARMS program in the CHR‐P subgroup at baseline (predictive model) and across the 2‐year follow‐up period (causal model).

T0‐T2 PANSS “Disorganization” factor score: Baseline predictors (*n* = 94)	B	SE	β	p	95% CI for B		VIF	
					Lower	Upper		
Constant	0.842	1.710	—	0.623	−2.546	4.230	—	*R* ^2^ = 0.311 *F*_[df=7]_ = 7.349 *p* = 0.**001**
T0 equivalent dose of chlorpromazine (mg/day)	0.625	0.196	0.266	**0.014** ^a^	0.236	1.014	1.157	
DUI	−0.020	0.007	−0.219	**0.048** ^a^	−0.034	−0.006	1.043	
T0 PANSS Positive symptoms score	−0.018	0.117	−0.014	0.875	−0.249	0.213	1.311	
T0 PANSS Negative symptoms score	−0.270	0.055	−0.418	**0.007** ^a^	−0.379	−0.116	1.188	
T0 PANSS Affect score	−0.170	0.080	−0.180	0.252	−0.330	−0.011	1.186	
T0 PANSS Resistance/Excitement−activity score	0.031	0.130	0.020	0.810	−0.226	0.289	1.181	
T0 PANSS G12 “Lack of judgment/insight” score	−0.604	0.320	−0.172	0.434	−1.237	0.030	1.366	

T0‐T2 PANSS “Disorganization” factor score: Effect of treatment on symptom changes (*n* = 94)	B	SE	β	p	95% CI for B		VIF	
					Lower	Upper		
Constant	−0.151	0.808	—	0.852	−1.756	1.453	—	*R* ^2^ = 0.551 *F*_[df=9]_ = 12.282 *p* = 0.**001**
T2 equivalent dose of chlorpromazine (mg/day)	0.076	0.205	0.027	0.713	−0.332	0.483	1.081	
T2 number of individual psychotherapy sessions	−0.050	0.024	−0.212	0.090	−0.097	−0.002	2.092	
T2 number of family psychoeducation sessions	0.080	0.040	0.194	**0.049** ^a^	0.001	0.159	1.914	
T2 number of case management sessions	0.006	0.005	0.080	0.286	−0.005	0.017	1.128	
T0‐T2 PANSS Positive symptoms score	0.196	0.080	0.210	0.096	0.037	0.355	1.477	
T0‐T2 PANSS Negative symptoms score	0.397	0.066	0.528	**0.007** ^a^	0.265	0.529	1.566	
T0‐T2 PANSS Affect score	0.127	0.097	0.113	0.195	−0.066	0.320	1.503	
T0‐T2 PANSS Resistance/Excitement−activity score	0.168	0.141	0.099	0.236	−0.112	0.449	1.373	
T0‐T2 PANSS G12 “Lack of judgment/insight” score	0.073	0.371	0.016	0.844	−0.663	0.809	1.315	

Abbreviations: 95% CI, 95% Confident Intervals for B; B, regression coefficient; CHR‐*P*, Clinical high Risk for Psychosis; df, degrees of freedom; DUI, Duration of Untreated Illness; F, statistic test value for linear regression; *p*, statistical significance; PANSS, Positive and Negative Syndrome Scale; PARMS, Parma At‐Risk Mental States; R^2^, *R*‐squared or coefficient of determination; SE, Standard Error; T0, baseline assessment time; T2, 2‐year assessment time; VIF, Variance Inflation factor;β, standardized regression coefficient.

^a^ Bonferroni corrected *p* values are reported. Statistically significant *p* values are in bold. Longitudinal analyses were conducted exclusively on participants that completed the 2‐year EIP treatment. As for potential multi‐collinearity of independent parameters, no variance inflation factor was greater than 5, suggesting moderate but no problematic correlations.

## 4 Discussion

Our results suggest that 2/3 of CHR‐P participants had an improvement in disorganization across the follow‐up. Although considered a treatment‐resistant dimension (Prasad et al. 2023), when early treated within CHR‐P programs, disorganization improves, reducing its potential negative effect on outcomes. Our CHR‐P/ID+ subjects showed specific clinical features: *shorter DUI* (supporting the impact of early detection strategies in prognosis) and lower baseline *anxious‐depressive* symptoms (this is less easily explainable, although Kline and co‐authors [2018] reported that baseline depression in CHR‐P was associated with reduced likelihood of clinical remission).

Longitudinal improvement in disorganization is related to improvement in other psychopathological dimensions (negative and affective domains) and in *cooperativeness* and *insight/judgment* subscores (supporting the relevance of treatment adherence and insight in improving prognosis) (Iuso et al. 2023). Finally, the contextual reduction of the PANSS total scores suggests the reliability of disorganization as a *general psychopathological severity index* over time.

Our results also suggest that the longitudinal mitigation of disorganization was predicted by baseline *AP* prescription, shorter DUI, intensity of *psychoeducation* sessions provided across the follow‐up, and longitudinal improvement in negative symptoms. Despite the guidelines' recommendations that advise against AP prescription in CHR‐P subjects, our findings support the potential effectiveness of AP in decreasing disorganization over time. This partially confirms their effectiveness on disorganization in first episode psychosis, through both direct effects on dopamine receptors and indirect improvements in global cognitive performance (Vita et al. 2022).

As for *limitations*, our findings remain generalizable to analogous CHR‐P samples. Second, our participants were recruited in a CHR‐P protocol not specifically focusing on disorganization. Third, although commonly used in early psychosis, our factor model for disorganization was identified primarily in schizophrenia. Fourth, no putative association between disorganized and neurocognitive characteristics was explored. Fifth, as temporal precedence cannot be disentangled, caution in interpreting our associations is needed. Sixth, the sampling from a single Italian CHR‐P service could introduce geographical/cultural bias. Seventh, we used an arbitrary cut‐off (30% decrease in PANSS disorganization) for subgroup operationalization. This could result in a loss of statistical power. Indeed, patients with higher baseline disorganization might be more likely to show a purely mechanical reduction ≥ 30, influencing group membership and correlating antipsychotic prescribing. However, no inter‐group differences in baseline PANSS disorganization severity levels were found (Table S4). Eighth, AP prescription was not randomly assigned and, although we used the equivalent dose of chlorpromazine, this could bring heterogeneity (i.e., patients with more severe conditions were more likely to receive AP treatment, and different primary symptoms led to the selection of different APs). At the same time, our regression analyses fitted only among participants who improved (CHR‐P/ID+) and this may be problematic for causal interpretation. Indeed, any variable potentially influencing the probability of being classified as CHR‐P/ID+ (e.g., baseline severity, adherence, clinicians' prescribing) may become a collider. Finally, the small sample size may compromise the interpretability of findings (e.g., evidence that psychotherapy and family intervention did not show significant effects should be considered with caution).

In conclusion, while some CHR‐P interventions are effective, more targeted treatments on disorganization in CHR‐P subjects are recommended.

## Author Contributions

**Lorenzo Pelizza** and **Emanuela Leuci:** study design and conceptualization. **Michele Occhionero, Arianna Biancalani**, and **Simona Pupo:** literature search. **Emanuela Leuci, Emanuela Quattrone**, and **Derna Palmisano:** data collection. **Lorenzo Pelizza, Michele Occhionero**, and **Arianna Biancalani:** formal analysis. **Lorenzo Pelizza, Michele Occhionero**, and **Arianna Biancalani:** writing – original draft. All authors: writing and editing – final version.

## Funding

The authors have nothing to report.

## Ethics Statement

Local ethics approval has been obtained for the investigation (AVEN Ethics Committee: protocol n. 559/2020/OSS*/AUSLPR). This examination was carried forward in agreement with the Declaration of Helsinki (1964) and its later amendments.

## Consent

All participants and their relatives (if minors) gave their written informed consent prior to their recruitment in the investigation.

## Conflicts of Interest

The authors declare no conflicts of interest.

## Supporting information


**Table S1:** Clinical assessment of our research.
**Table S2:** CFA indices of adjustment in the CHR‐P total sample (*n* = 180): exploring competitive models of two different PANSS “Disorganization” factor configurations proposed in the current literature.
**Table S3:** Specialized treatment components of the PARMS program.
**Table S4:** Baseline sociodemographic and clinical characteristics in the two CHR‐P groups (*n* = 180).
**Figure S1:** CHR‐P participants with improvement in PANSS “Disorganization” factor score across the 2‐year follow‐up period.
**Table S5:** Longitudinal association between disorganization and other clinical parameters in the CHR‐P subgroup with improvement in PANSS “Disorganization” factor score across the 2‐year follow‐up period (*n* = 94).

## Data Availability

The data supporting the results of this investigation are available on reasonable request. The data are not publicly available due to ethical or privacy restrictions.

## References

[appy70021-bib-0001] AAA . 2023. “AAA.”

[appy70021-bib-0002] AAA . 2025. “AAA.”

[appy70021-bib-0003] American Psychiatric Association . 2013. Diagnostic and Statistical Manual of Mental Disorders, 5th Edition. American Psychiatric Publishing.

[appy70021-bib-0004] Bleuler, E. 1911. Dementia Praecox Oder Gruppe der Schizophrenien. Verlag. 10.1192/bjp.149.5.661 3545358

[appy70021-bib-0005] First, M. B. , J. B. Williams , R. S. Karg , and R. L. Spitzer . 2016. “Structured Clinical Interview for DSM‐5 Disorders—Clinical Version (SCID‐5‐CV).” New York State Psychiatric Institute.

[appy70021-bib-0006] Iuso, S. , M. Severo , N. Trotta , et al. 2023. “Improvements in Treatment Adherence After Family Psychoeducation in Patients Affected by Psychosis: Preliminary Findings.” Journal of Personalized Medicine 13: 1437. 10.3390/jpm13101437. 37888048 PMC10608243

[appy70021-bib-0007] Lin, C. H. , H. S. Lin , S. C. Lin , C. C. Kuo , F. C. Wang , and Y. H. Huang . 2018. “Early Improvement in PANSS‐30, PANSS‐8, and PANSS‐6 Scores Predicts Ultimate Response and Remission During Acute Treatment of Schizophrenia.” Acta Psychiatrica Scandinavica 137: 98–108. 10.1111/acps.12849. 29280500

[appy70021-bib-0008] Prasad, K. , J. Rubin , S. Iyengar , and J. Cape . 2023. “Global Network Disorganization Underlying Psychosis High Risk States.” Schizophrenia Research 255: 67–68. 10.1016/j.schres.2023.03.033. 36965361

[appy70021-bib-0009] Ver Hoef, J. M. 2012. “Who Invented the Delta Method?” American Statistician 66: 124–127. 10.1080/00031305.2012.687494.

[appy70021-bib-0010] Vita, A. , W. Gaebel , A. Mucci , et al. 2022. “European Psychiatric Association Guidance on Treatment of Cognitive Impairment in Schizophrenia.” European Psychiatry 65: e57. 10.1192/j.eurpsy.2022.2315. 36059103 PMC9532218

