# Supplementary material for: Clinical Factors Influencing Improvement in Disorganization in Clinical High‐Risk for Psychosis: Preliminary Findings From a 2‐Year Follow‐Up
Source: Asia Pac Psychiatry. 2026 Apr 8;18(2):e70021. doi: 10.1111/appy.70021 (PMC13060276; doi:10.1111/appy.70021)
Supplement: Supplementary file 1 — Table S1: Clinical assessment of our research. Table S2: CFA indices of adjustment in the CHR‐P total sample (n = 180): exploring competitive models of two different PANSS “Disorganization” factor configurations proposed in the current literature. Table S3: Specialized treatment components of the PARMS program. Table S4: Baseline sociodemographic and clinical characteristics in the two CHR‐P groups (n = 180). Figure S1: CHR‐P participants with improvement in PANSS “Disorganization” factor score across the 2‐year follow‐up period. Table S5: Longitudinal association between disorganization and other clinical parameters in the CHR‐P subgroup with improvement in PANSS “Disorganization” factor score across the 2‐year follow‐up period (n = 94). [file APPY-18-e70021-s001.docx]

Table S1 – Clinical assessment of our research.

| In the clinical assessment of this investigation, we used the GAF, the PANSS, and the CAARMS.  +) The *CAARMS* mainly explores attenuated psychopathology. Specifically, its “*Positive Symptoms*” score defines CHR-P thresholds. PARMS members were trained to conduct CAARMS interviews at baseline and used the authorized Italian version.  +) The *PANSS* evaluates psychopathology in subjects with psychosis, including early psychosis and CHR-P. Specifically, after having controlled for goodness of fit of the 2 different PANSS “Disorganization” factor models currently proposed in the literature using a Confirmatory Factor Analysis (see Table S2 in the Supplementary Materials for details), the 8-item solution (including PANSS N5 “Difficulty in abstract thinking”, P2 “Conceptual disorganization”, G5 “Mannerisms and posturing”, N7 “Stereotyped thinking”, G11 “Poor attention”, G10 “Disorientation”, G15 “Preoccupation”, and G13 “Disturbance of volition” items) proposed by Shafer and Dazzi (2019) was selected for our statistical analysis. In our intention, CFA was necessary to choose the “Disorganization” factor that best fitted our sample. However, we also used the complete 5-factor model indicated in their meta-analysis on the PANSS structure including the following dimensions in addition to the “Disorganization” domain: “Positive Symptoms”, “Negative Symptoms”, “Affect” (anxious-depressive), and “Resistance/Excitement-Activity”. According to the authors, the items defining the “Disorganization” factor were relatively well-defined in the meta-analyses and did not overlap with the items included in other domains (especially with negative symptoms).  +) The *GAF* assesses socio-occupational and daily clinical functioning in patients with early psychosis and at CHR-P.  Furthermore, an “ad-hoc” chart including information on socio-demographic, clinical, and treatment features was also completed. Specifically, the “Duration of Untreated Illness” (DUI) was intended as the time interval (in weeks) between the beginning of any psychopathological features in need of treatment and the first specialized psychosocial/psychopharmacological therapy and was collected by the patient and at least one significant parent.  Finally, the DSM-5 diagnosis was made at entry by two trained PARMS staff members with the SCID-5 (Structured Clinical Interview for DSM-5 disorders) (First et al., 2016).  All instruments and information on AP dose and intensity of PARMS psychosocial treatments were collected both at presentation and every 12 months during the 2-year observation time. |
| --- |

Note – GAF = Global Assessment of Functioning; PANSS = Positive And Negative Syndrome Scale; CAARMS = Comprehensive Assessment of At-Risk Mental States; PARMS = Parma At-Risk Mental States program; CHR-P = Clinical High Risk for Psychosis; DSM-5 = Diagnostic and Statistical Manual of mental disorders, 5^th^ Edition.

Table S2 - CFA indices of adjustment in the CHR-P total sample (n = 180): exploring competitive models of two different PANSS “Disorganization” factor configurations proposed in the current literature.

| PANSS “disorganization” factor models | χ^2^  (df; p) | CFI | TLI | RMSEA | SRMR | AIC |
| --- | --- | --- | --- | --- | --- | --- |
| T0  8-item factor model  5-factor model  T1  8-item factor model  5-factor model | 46.194  (20;.0001)  5.993  (5;.307)  67.886  (20;.0001)  15.638  (5;.008) | .990  .894  .945  .914 | .981  .851  .891  .880 | .126  .473  .132  .140 | .051  .098  .078  .109 | 1898.166  2948.676  1837.721  2687.243 |

Note - CFA = Confirmatory Factor Analysis; PANSS = Positive And Negative Syndrome Scale; CHR-P = Clinical High Risk for Psychosis; χ^2^ = Chi-squared test value; df = degrees of freedom; p = statistical significance; T0 = baseline assessment time; T1 = 1-year assessment time; CFI = Comparative Fit Index; TLI = Tucker-Lewis Index; RMSEA = Root Mean Square Error of Approximation; SRMR = Standardized Root Mean Square Residual; AIC = Akaike Information Criterion; 8-item “Disorganization” factor model including PANSS P2, N5, N7, G5, G10, G11, G13, and G15 items (Shafer & Dazzi, 2019); 5-item factor model including PANSS P2, P5, G9, G11, and G13 items (Yang et al., 2018).

Shafer, A., & Dazzi, F. (2019). Meta-analysis of the Positive and Negative Syndrome Scale (PANSS) factor structure. Journal of Psychiatric Research, 115, 113-120. <https://doi.org/10.1016/j.jpsychires.2019.05.008>.

Yang, Z., Lim, K., Lam, M., Keefe, R., & Lee, J. (2018). Factor structure of the positive and negative syndrome scale (PANSS) in people at ultra-high risk (UHR) for psychosis. Schizophrenia Research, 201, 85–90. <https://doi.org/10.1016/j.schres.2018.05.024>.

Table S3 – Specialized treatment components of the PARMS program.

| Within the PARMS protocol, CHR-P individuals were assigned to a multi-disciplinary team (consisting of a clinical psychologist, an early rehabilitation case manager, and a psychiatrist) within 4 weeks.  In accordance with current official guidelines on “Early Intervention in Psychosis” (EIP) (Schmidt et al., 2015; RER, 2024), *Antipsychotic* (AP) drug prescription should be proposed when individuals at CHR-P (a) showed a sudden decline in daily functioning, (b) had a rapid escalation to overt psychotic symptoms, (c) showed an immediate risk of suicide or severe violence, or (d) did not adequately respond to any other psychosocial interventions. As a first-line pharmacological treatment, low-dose second-generation AP medication was indicated (Raballo et al., 2021). Antidepressant and benzodiazepine medication could be prescribed for anxiety, depressive symptoms, and/or insomnia.  *Individual psychotherapy* was shaped on the model suggested by Van der Gaag and colleagues (2012) for CHR-P individuals. At least 15 sessions per CHR-P subject (each lasting 60 minutes) were offered during the first year of treatment (Azzali et al., 2022). Booster sessions were also provided in the second year according to specific clinical needs.  *Family psychoeducation* was adapted on the model developed by McFarlane and co-workers (2012) for CHR-P individuals. In the first year of treatment, at least 10 psychoeducational sessions were offered to each family (Pelizza et al., 2019). Booster sessions could also be provided in the second year according to specific family problems or clinical needs.  Finally, each individual/family had a dedicated *case-manager* to coordinate all interventions that have been planned, especially those promoting a recovery-oriented early rehabilitation (such as “patient-tailored” care pathways specifically designed for encouraging functional recovery, social inclusion and job, also using models inspired by supported employment and community care) (Pelizza et al., 2020; Ficarelli et al., 2021). At least 24 sessions per CHR-P subject (each lasting 60 minutes) were provided along the 2 years of follow-up. |
| --- |

Note. PARMS = Parma At-Risk Mental States; CHR-P = Clinical High Risk for Psychosis.

Schmidt SJ, Schultze-Lutter F, Schimmelmann BG, Maric NP, Salokangas RK, Riecher-Rössler A, van der Gaag M, Meneghelli A, Nordentoft M, Marshall M, Morrison A, Raballo A, Klosterkötter J, Ruhrmann S (2015) EPA guidance on the early intervention in clinical high-risk states of psychoses. Eur Psychiatry 30: 388-404. <https://doi.org/10.1016/j.eurpsy.2015.01.013>.

Regione Emilia-Romagna (RER) (2024) Raccomandazioni regionali per la promozione della salute e del benessere in persone all’esordio psicotico, II edition. Centro Stampa della Regione Emilia-Romagna, Bologna.

Raballo A, Poletti M, Preti A (2021) Antipsychotic treatment in clinical high risk for psychosis: protective, iatrogenic or further risk flag? Aust N Z J Psychiatry 55: 442-444. https://doi.org/10.1177/0004867420984836.

Van der Gaag M, Nieman DH, Rietdijk J, Dragt S, Ising HK, Klaassen RM, Koeter M, Cuijpers P, Wunderink L, Linszen DH (2012) Cognitive behavioral therapy for subjects at ultrahigh risk for developing psychosis: a randomized controlled clinical trial. Schizophr Bull 38: 1180-1188. <https://doi.org/10.1093/schbul/sbs105>.

Azzali S, Pelizza L, Scazza I, Paterlini F, Garlassi S, Chiri LR, Poletti M, Pupo S, Raballo A (2022) Examining subjective experience of aberrant salience in young individuals at ultra-high risk (UHR) of psychosis: a 1-year longitudinal study. Schizophr Res 241: 52-58. <https://doi.org/10.1016/j.schres.2021.12.025>.

McFarlane WR, Lynch S, Melton R (2012) Family psychoeducation in clinical high risk and first-episode psychosis. Adolesc Psychiatry 2: 182-194. <https://doi.org/10.2174/2210676611202020182>.

Pelizza L, Azzali S, Garlassi S, Scazza I, Paterlini F, Chiri LR, Poletti M, Pupo S, Raballo A (2019) Examining subjective experience of social cognition in early psychosis: validation of the Italian version of the GEOPTE scale in an adolescent and young adult clinical sample. J Psychopathol 25: 220-230. <https://old.jpsychopathol.it/wp-content/uploads/2019/12/06_Pelizza-1.pdf>.

Pelizza L, Leuci E, Landi G, Quattrone E, Azzali S, Pelosi A, Ceroni P, Soncini C, Daolio MC, Dall’Aglio R, Paulillo G, Pellegrini C, Raballo A, Pellegrini P (2020) The “Personal Health Budget” intervention model in early psychosis: preliminary findings from the Parma experience. J Psychopathol 26: 209-217. <https://doi.org/10.36148/2284-0249-359>.

Ficarelli ML Troisi E, Vignali E, Artoni S, Franzini MC, Montanaro S, Andreoli MV, Marangoni S, Ciampà E, Erlicher D, Pupo S, Pelizza L (2021) Implementing individual and placement support for patients with severe mental illness: findings from the real world. J Psychopathol 27: 71-80. [https://doi:org/10.36148/2284-0249-346](rewritten://37d9e666-dade-410a-852c-b24c95d161d6).

Table S4 – Baseline sociodemographic and clinical characteristics in the two CHR-P groups (n = 180).

| Variables | CHR-P/ID+  (n = 111) | CHR-P/ID-  (n = 69) | Χ^2^/z | p |
| --- | --- | --- | --- | --- |
| Gender (males)  Ethnic group (white Caucasian)  Migrant Status  Age (at entry)  Education (in years)  Civil status (single)  Living status (with parents)  NEET  DUI (in weeks)  Past hospitalization  Past attempted suicide  Past specialist contact  Family history of psychosis  Current substance abuse  *CHR-P subgroups*  APS BLIPS  Genetic vulnerability  *DSM-5 diagnoses*  Depressive disorder  Psychotic disorder NOS  Schizotypal personality disorder  Brief psychotic disorder  Anxiety disorder  Borderline personality disorder  Obsessive-compulsive disorder  Eating disorder  *PANSS scores*  Positive symptoms  Negative symptoms  Disorganization  Affect  Resistance/Excitement-activity  Total score  G8 “Uncooperativeness”  G12 “Lack of judgment/insight”  GAF score  Baseline AP prescription  Equivalent dose of risperidone (mg/day)  Baseline AD prescription  Baseline MS prescription  Baseline BDZ prescription  2-year service disengagement incidence rate  2-year psychosis transition rate | 54 (48.6%)  97 (87.4%)  14 (12.6%)  19.62±3.89  11.31±2.56  108 (97.3%)  104 (93.7%)  31 (27.9%)  38.89±40.01  16 (14.4%)  49 (44.1%)  12 (10.8%)  36 (32.4%)  18 (16.2%)  87 (78.4%)  18 (16.2%)  6 (5.4%)  38 (34.2%)  21 (18.9%)  17 (15.3%)  13 (11.7%)  9 (8.1%)  7 (6.3%)  4 (3.6%)  2 (1.8%)  10.61±3.71  20.30±8.24  16.46±5.74  14.45±5.19  6.99±2.95  70.95±18.74  1.64±1.05  2.14±1.35  48.51±8.66  59 (53.2%)  2.84±2.08  25 (22.5%)  9 (8.1%)  23 (20.7%)  17 (15.3%)  16 (14.4%) | 36 (52.2%)  62 (89.9%)  14 (20.3%)  19.38±3.65  11.35±2.24  68 (98.5%)  65 (94.2%)  24 (34.8%)  58.42±58.59  12 (17.4%)  34 (49.3%)  7 (10.1%)  23 (33.3%)  13 (18.8%)  53 (76.8%)  12 (17.4%)  4 (5.7%)  24 (34.8%)  8 (11.6%)  9 (13.0%)  11 (15.9%)  5 (7.2%)  6 (8.7%)  4 (5.8%)  5 (7.2%)  11.00±3.89  17.65±6.30  14.36±4.37  16.91±4.86  7.70±3.60  70.17±15.03  1.55±1.12  2.40±1.48  49.07±8.58  33 (47.8%)  2.34±1.58  18 (26.1%)  8 (11.6%)  18 (26.1%)  10 (14.5%)  13 (18.8%) | .212  .251  1.909  -.492  -.264  .031  .198  .219  -2.344  .287  .451  .020  .016  .206  .060  .042  .002  3.212  -.591  -1.526  -1.697  -2.736  -.668  -.407  -.987  .915  -.210  .483  -.950  .297  .605  .697  .023  .142 | .646  .616  .167  .623  .792  .999  .513  .649  **.019**  .592  .502  .888  .900  .650  .806  .837  .962  .865  .555  .127  .090  **.009**  .504  .684  .324  .360  .834  .487  .342  .589  .437  .404  .881  .776 |

Note. CHR-P = Clinical High Risk for Psychosis; ID = Improvement in PANSS “Disorganization” factor score across the follow-up; CHR-P/ID+ = CHR-P participants with ID; CHR-P/ID- = CHR-P participants without ID; NEET = Not in Education, Employment, or Training; DUI = Duration of Untreated Illness; APS = Attenuated Psychotic Symptoms, BLIPS = Brief Limited Intermittent Psychotic Symptoms; GRFD = Genetic Risk Functioning Deterioration syndrome; NOS = Not Otherwise Specified; GAF = Global Assessment of Functioning; AP = Antipsychotic; AD = Antidepressant; MS = Mood Stabilizer; BDZ = Benzodiazepine; p = statistical significance. Frequencies (and percentages), mean ± standard deviation, Chi-Square (X^2^) test and Mann-Whitney test (z) values are reported. Statistically significant p values are in bold.

Figure S1 – CHR-P participants with improvement in PANSS “Disorganization” factor score across the 2-year follow-up period.

180 CHR-P participants

10 CHR-P/ID-

(service disengagement)

111 CHR-P/ID+

17 CHR-P/ID+

(service disengagement)

69 CHR-P/ID-

59 CHR-P/ID-

94 CHR-P/ID+

Note. CHR-P = Clinical High Risk for Psychosis; ID = Improvement in PANSS “Disorganization” factor score across the follow-up period; CHR-P/ID+ = CHR-P participants with ID; CHR-P/ID- = CHR-P participants without ID; T1 = 1-year assessment time; T2 = 2-year assessment time.

Table S5 – Longitudinal association between disorganization and other clinical parameters in the CHR-P subgroup with improvement in PANSS “Disorganization” factor score across the 2-year follow-up period (n=94).

| Variable | T0-T2 PANSS  “Disorganization” factor score (ρ/z) | p |
| --- | --- | --- |
| Gender  Ethnic group (white Caucasian)  Migrant status  Age (at entry)  Education (in years)  DUI (in weeks)  Family history of psychosis  Past specialist contact  Substance abuse (at entry)  *CHR-P subgroups*  APS  BLIPS  *T0-T2 PANSS scores*  Positive symptoms  Negative symptoms  Affect  Resistance/Excitement-Activity  Total score  G8 “Uncooperativeness”  G12 “Lack of judgment/insight”  T0-T2 GAF score | -1.735  -.292  .001  .209  .008  -.275  -1.220  -1.006  -.258  -.689  -.475  .010  .520  .369  .296  .689  .232  .226  -.044 | .083  .771  .999  .070  .944  **.032**  .223  .314  .796  .491  .635  .931  **.001**^*^  **.001**^*^  **.009**^*^  **.001**^*^  **.019**^*^  **.023**^*^  .740 |

Note. CHR-P = Clinical High Risk for Psychosis; PANSS = Positive And Negative Syndrome Scale; Duration of Untreated Illness; APS = Attenuated Psychotic Symptoms; BLIPS = Brief Limited Intermittent Psychotic Symptoms; T0 = baseline assessment time; T1 = 1-year assessment time; T2 = 2-year assessment time; GAF = Global Assessment of Functioning; p = statistical significance. Spearman rank correlation (ρ) and Mann-Whitney U test (z) values are reported. ^*^Bonferroni corrected p values are reported. Statistically significant p values are in bold. Longitudinal analyses were conducted exclusively on participants that completed the 2-year EIP treatment.
